# The prevalence and anatomy of parathyroid glands: a meta-analysis with implications for parathyroid surgery

**DOI:** 10.1007/s00423-019-01751-8

**Published:** 2019-02-14

**Authors:** Dominik Taterra, Linda M. Wong, Jens Vikse, Beatrice Sanna, Przemysław Pękala, Jerzy Walocha, Roberto Cirocchi, Krzysztof Tomaszewski, Brandon Michael Henry

**Affiliations:** 1 International Evidence-Based Anatomy Working Group, 12 Kopernika St., 31-034 Kraków, Poland; 2 Department of Anatomy, Jagiellonian University Medical College, Kraków, Poland; 3 Department of Surgery, Stavanger University Hospital, Stavanger, Norway; 4 Department of Surgical Sciences, University of Cagliari, Monserrato, Italy; 5 Department of Surgical Sciences, Radiology and Dentistry, University of Perugia, Perugia, Italy

**Keywords:** Parathyroid glands, Meta-analysis, Anatomy, Hyperparathyroidism, Parathyroidectomy

## Abstract

**Purpose:**

The anatomy of parathyroid glands (PTG) is highly variable in the population. The aim of this study was to conduct a systematic analysis on the prevalence and location of PTG in healthy and hyperparathyroidism (HPT) patients.

**Methods:**

An extensive search of the major electronic databases was conducted to identify all studies that reported relevant data on the number of PTG per patient and location of PTG. The data was extracted from the eligible studies and pooled into a meta-analysis.

**Results:**

The overall analysis of 26 studies (*n* = 7005 patients; *n* = 23,519 PTG) on the number of PTG showed that 81.4% (95% CI 65.4–85.8) of patients have four PTG. A total of 15.9% of PTG are present in ectopic locations, with 11.6% (95% CI 5.1–19.1) in the neck and 4.3% (95% CI 0.7–9.9) in mediastinum. The subgroup analysis of ectopic PTG showed that 51.7% of ectopic PTG in the neck are localized in retroesophageal/paraesophageal space or in the thyroid gland. No significant differences were observed between the healthy and HPT patients and cadaveric and intraoperative studies.

**Conclusions:**

Knowledge regarding the prevalence, location, and anatomy of PTG is essential for surgeons planning for and carrying out parathyroidectomies, as any unidentified PTG, either supernumerary or in ectopic location, can result in unsuccessful treatment and need for reoperation.

**Electronic supplementary material:**

The online version of this article (10.1007/s00423-019-01751-8) contains supplementary material, which is available to authorized users.

## Introduction

**Fig. 1:**
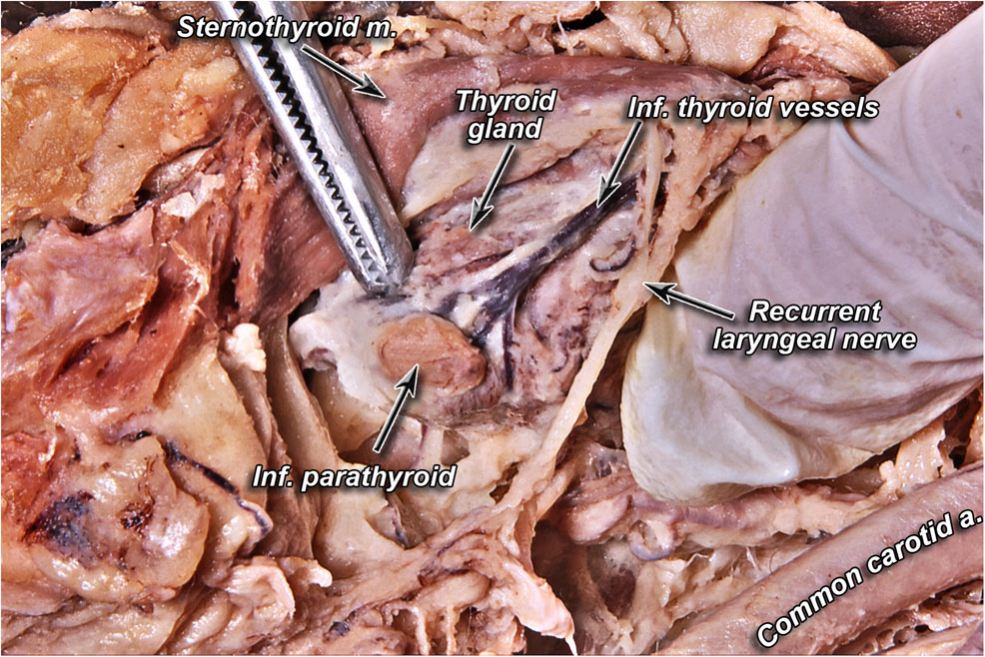
Anatomical relationships of parathyroid gland

Parathyroid glands (PTG) are nodular structures that are usually located along the posterior wall of the thyroid (Fig. 1). Their product, parathyroid hormone (PTH), plays an essential role in calcium homeostasis in the organism. Elevation of PTH can occur due to either overproduction by an adenomatous, hyperplastic, or rarely carcinomatous gland in primary hyperparathyroidism (PHPT) or due to hypocalcemia in secondary hyperparathyroidism (SHPT). Hypocalcemia may result from chronic kidney disease or malabsorption. The standard treatment for symptomatic patients with PHPT is surgical excision of transformed glands. If the underlying cause of hypocalcemia in patients with SHPT cannot be addressed or if patients are refractory to pharmacological therapy, a parathyroidectomy is the treatment of choice [1].

The anatomy of PTG is highly variable in the population. Classically, four PTG are present, but previous research reported fewer and cases of up to 12 glands per patient [2]. Parathyroid glands derive from endodermal tissue. Embryologically, the two superior glands descend from the fourth pharyngeal pouch and the two inferior glands from the third pharyngeal pouches [3]. The superior glands are usually located on the upper pole of the thyroid, a short distance caudally from the intersection of the recurrent laryngeal nerve and inferior thyroid artery. Since the traveled distance of superior glands is shorter, their location is often more constant than the inferior glands [3]. The latter descend along with the thymus, which also originates from the third pharyngeal pouch. Due to this fact, the glands can often be found along the descent pathway in the neck or mediastinum [3]. Their most frequent location is the inferior pole of the thyroid, below the superior PTG.

Bilateral neck exploration is the classical approach to parathyroidectomy. Preoperative identification of PTG is of crucial importance, especially in patients with PHPT requiring excision of only one or few PTG and allows for a less invasive approach. Ultrasonography (USG), technetium-99m scintigraphy, or computed tomography are used to identify PTG preoperatively. Moreover, intraoperative PTH assay ensures that all PTG causing hyperparathyroidism are removed during surgery. Substantial research has been done to untangle the variable prevalence and location of PTG, but the results are inconsistent. Knowledge regarding the prevalence, location, and anatomy of PTG is essential for surgeons planning for and carrying out parathyroidectomies, as any unidentified PTG, either supernumerary or those in an ectopic location, can result in unsuccessful treatment and the need for reoperation. Moreover, the preservation of PTG is of utmost importance during thyroidectomies. Therefore, the aim of this study was to conduct a systematic analysis on the prevalence, location, and morphometric data of PTG in both healthy patients and those with hyperparathyroidism (HPT).

## Materials and methods

### Search strategy

All major electronic databases, such as PubMed, Embase, ScienceDirect, SciELO, BIOSIS, and Web of Science were searched up to August 2018 for any studies reporting relevant data on the PTG. The following search terms were used: parathyroids OR parathyroid gland OR accessory parathyroid OR glandula parathyreoidea OR parathyroid bodies. The search was not limited by any date or language restrictions. The Preferred Reporting Items for Systematic Reviews and Meta-Analyses (*PRISMA*) guidelines were strictly followed throughout this study (Supplement 1).

### Eligibility assessment

Two independent reviewers conducted eligibility assessment of any potential article. Any studies that were peer-reviewed and included data about the number of PTG per patient, location of PTG, or morphology of PTG were included into the meta-analysis. Studies that were conference abstracts, letters to editor, reviews, and studies with incomplete data were excluded. The articles published in languages other than English were translated by medical professionals, and their eligibility was assessed. Any dispute in the assessment of the eligibility was resolved by uniform consensus.

### Data extraction

Two independent reviewers extracted data from eligible articles. Data on the study type, number of PTG per patient, and location of PTG was extracted. Patients were subdivided into healthy and HPT groups. Patients who did not have any pathology of PTG were considered healthy. The PTG were classified into orthotopic, neck, or mediastinum based on location and later analyzed within each group. The superior PTG were considered orthotopic when located on the posterior aspect of the middle to upper pole of thyroid gland, while considered inferior PTG when located lateral to the inferior pole of the thyroid gland. Specific locations of ectopic glands in the neck and mediastinum were also noted when available.

### Statistical analysis

Software MetaXL 5.4 by EpiGear International Pty Ltd. (Wilston, Queensland, Australia) was used to conduct statistical analysis. The pooled prevalence estimates (PPE) were calculated using a random effects model. The heterogeneity of the included studies was assessed with chi^2^ test and *I*^2^ statistic. Significant heterogeneity was determined if Cochrane Q *p* value < 0.10 [4]. The following intervals for *I*^2^ statistic were used: 0–40%—“might not be important,” 30–60%—“might indicate moderate heterogeneity,” 50–90%—“may indicate substantial heterogeneity,” and 75–100%—“may represent considerable heterogeneity” [4].

The data was divided into several subgroups. Whenever possible, the type of study, patient’s health characteristic (healthy/HPT), and geographical location were analyzed separately. Moreover, sensitivity analysis by exclusion of studies with sample size smaller than 500 was performed to further investigate the source of heterogeneity. Confidence intervals were used to compare two groups, with any overlap as an indication of statistically insignificant difference [5].

## Results

### Study identification

**Fig. 2:**
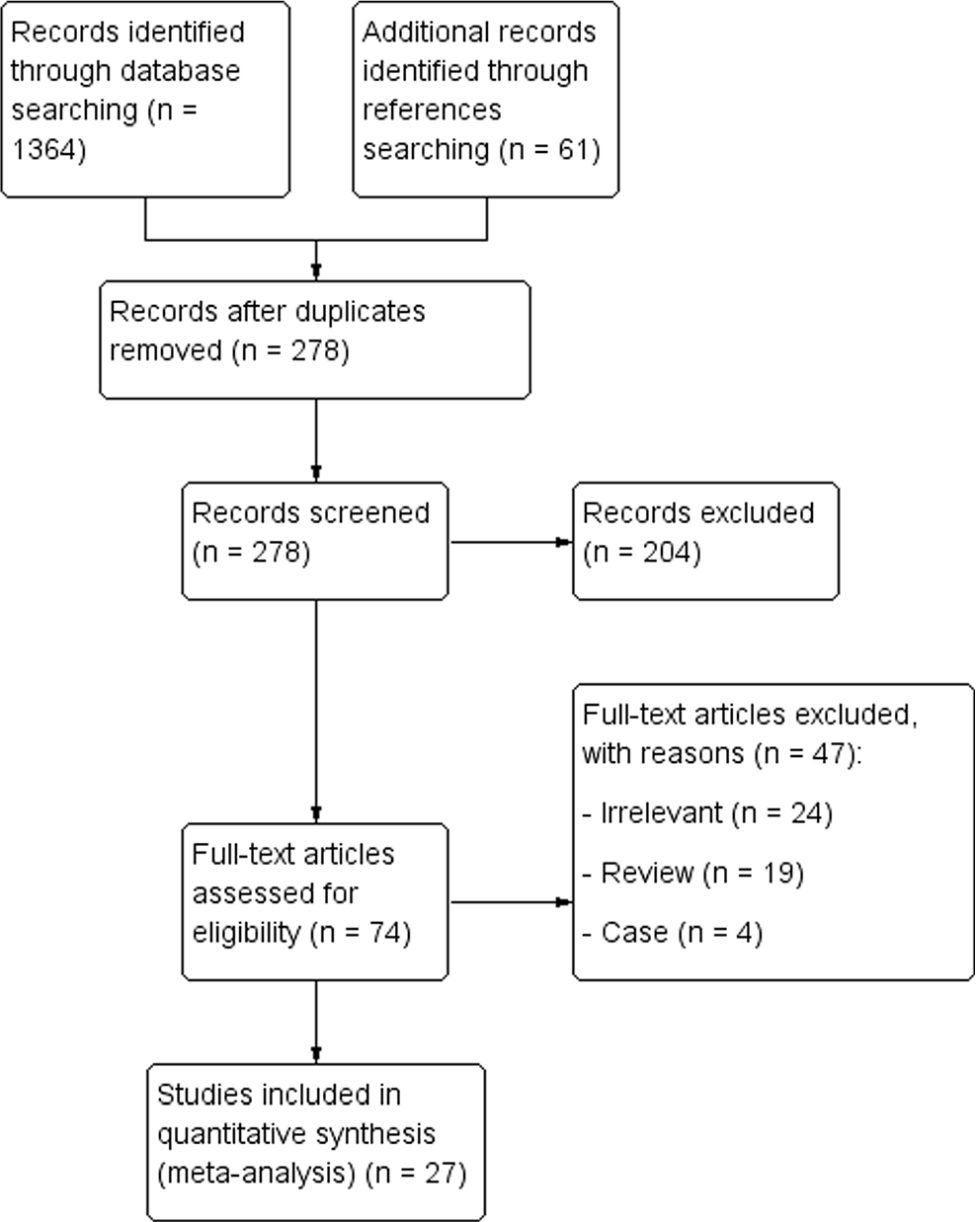
Study identification flowchart

The flow of articles through the study is presented in Fig. 2. The initial search identified 1364 entries. After exclusion of duplicates and initial screening, 74 studies were analyzed by full text.

Finally, a total of 27 articles were included into the meta-analysis.

### Characteristics of the included studies

Twenty-seven studies [2, 6–31] (*n* = 7106 patients, *n* = 23,519 PTG) analyzing the prevalence, location, or morphology of PTG were included. The dates of the included studies ranged from 1916 to 2016. Eleven studies originated from Europe, 6 from North America, 4 from South America, 5 from Asia, and 1 from Australia, and a total of 12 countries. Twelve studies analyzed healthy patients, and 10 studies analyzed patients with HPT, 2 with MEN1 and 2 with various thyroid diseases (Table 1).

**Table 1:** Characteristics of included studies

Study ID	Country	Type of study	Pathology	Number of patients	Number of analyzed PTG
Abboud 2008 [4]	Lebanon	Intraoperative	Various thyroid disease	574	2219
Akerstrom 1984 [5]	Sweden	Cadaveric	Healthy	503	2032
Alveryd 1968 [6]	Sweden	Cadaveric	Healthy	352	1405
Arnalsteen 2003 [7]	France	Intraoperative	MEN1	79	340
Botelho 2004 [8]	Brazil	Cadaveric	Healthy	19	76
Butterworth 1998 [9]	UK	Intraoperative	SHPT	60	241
de Andrade 2014 [10]	Brazil	Intraoperative	SHPT	166	664
Edis 1987 [11]	Australia	Intraoperative	SHPT	20	73
Ghandur 1986 [12]	USA	Cadaveric	Healthy	166	502
Gilmour 1938 [13]	UK	Cadaveric	Healthy	428	1713
Gomes 2007 [14]	Brazil	Intraoperative	SHPT	35	143
Heinbach 1933 [15]	USA	Cadaveric	Healthy	25	86
Hellman 1998 [16]	Sweden	Intraoperative	MEN1	50	206
Hibi 2002 [17]	Japan	Intraoperative	SHPT	822	
Hojaij 2011 [18]	Brazil	Cadaveric	Healthy	56	220
Kawata 2008 [19]	Japan	Intraoperative	SHPT	44	163
Lappas 2012 [20]	Greece	Cadaveric	Healthy	942	3796
Milas 2003 [21]	USA	Intraoperative	PHPT	828	3250
Nanka 2006* [22]	Czech Republic	Cadaveric	Healthy	101	280
Numano 1998 [23]	Japan	Intraoperative	SHPT	570	2377
Okada 2016 [24]	Japan	Intraoperative	SHPT	131	457
Pacini 1983 [25]	Italy	Intraoperative	PHPT and SHPT	42	163
Périé 2005 [26]	France	Intraoperative	SHPT	20	80
Pool 1916 [27]	USA	Cadaveric	Healthy	25	60
Prazenica 2015 [28]	Czech Republic	Intraoperative	Various thyroid disease	788	1937
Pyrtek 1964 [29]	USA	Cadaveric	Healthy	100	391
Wang 1976 [30]	USA	Cadaveric	Healthy	160	645

*SHPT* secondary hyperparathyroidism, *PHPT* primary hyperparathyroidism

*Study included only in location analysis

### Number of parathyroid glands per patient

**Fig. 3:**
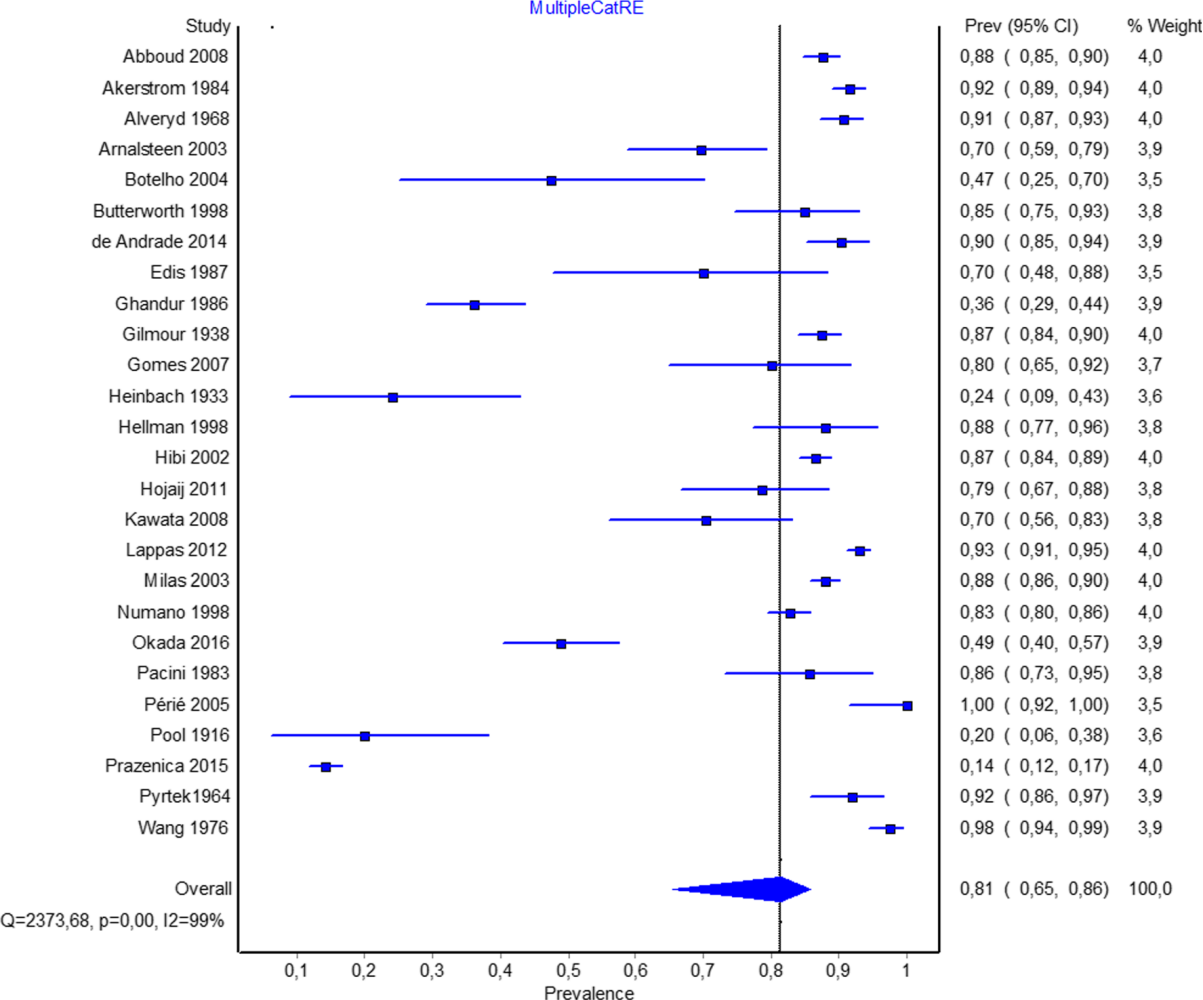
Forrest plot for overall pooled prevalence rate of patients with four parathyroid glands

The overall analysis of 26 studies (*n* = 7005 patients) on the number of parathyroid glands showed that 81.4% (95% CI 65.4–85.8) have four PTG (Fig. 3). Sensitivity analysis showed no significant differences compared with the overall analysis with high heterogeneity persisting. The subgroup analysis of 14 intraoperative studies (*n* = 3399 patients) revealed that 82.2% (95% CI 59.1–92.7) of patients have four PTG (Table 2). Similarly, 11 cadaveric studies (*n* = 2776 patients) showed that majority of patients have four PTG. Subgroup analysis of the healthy group based on geographical location revealed smaller prevalence of patients with four PTG both in North (62.5%; 95% CI 17.3–95.8) and South America (63.4%; 95% CI 32.1–93.7) than in Europe (90.9%; 95% CI 87.7–93.6); however, the differences were not significant. Overall, 4.9% and 6.3% of healthy and HPT patients, respectively, have five or more PTG (Table 2). Table 3 presents data on the subgroup analysis of the number of PTG in studies reporting prevalence of up to 11 PTG.

**Table 2:** Number of parathyroid glands per patient

Subgroup		Number of studies (number of patients)	PPE of patients with 0 PTG: % (95% CI)	PPE of patients with 1 PTG: % (95% CI)	PPE of patients with 2 PTG: % (95% CI)	PPE of patients with 3 PTG: % (95% CI)	PPE of patients with 4 PTG: % (95% CI)	PPE of patients with 5 PTG or more: % (95% CI)	*I*^2^: % (95% CI)	Cochran’s Q, *p* value
Overall		26 (7005)	0.5 (0–2.7)	1.0 (0.0–4.0)	2.6 (0.0–7.9)	9.1 (2.8–16.6)	81.4 (65.4–85.8)	5.4 (0.8–11.8)	98.9 (98.8–99.0)	< 0.001
Sensitivity		8 (5455)	0.1 (0.0–4.6)	0.3 (0.0–5.6)	1.7 (0.0–9.8)	7.1 (0.0–20.3)	86.4 (61.3–96.8)	4.4 (0.0–15.7)	99.6 (99.6–99.7)	< 0.001
Type of study	Intraoperative	14 (3399)	0.5 (0.0–5.2)	1.0 (0.0–7.0)	2.4 (0.0–10.3)	7.3 (0.0–21.3)	82.2 (59.1–92.7)	6.6 (0.0–17.7)	99.2 (99.0–99.3)	< 0.001
	Cadaveric	11 (2776)	0.5 (0.0–3.1)	1.2 (0.0–4.6)	4.1 (0.2–10.9)	9.7 (2.8–18.2)	79.6 (62.7–85.3)	4.9 (0.4–11.9)	97.5 (96.6–98.2)	< 0.001
Geographical origin	Europe	4 (2225)	0.0 (0.0–0.4)	0.0 (0.0–0.4)	0.2 (0.0–0.8)	3.9 (2.2–6.2)	90.9 (87.7–93.6)	4.9 (2.9–7.3)	82.0 (53.5–93.0)	0.001
	North America	5 (476)	1.2 (0.0–18.8)	2.6 (0.0–23.8)	12.5 (0.0–45.2)	18.3 (0.0–54.2)	62.5 (17.3–95.8)	2.9 (0.0–24.7)	98.5 (97.8–99.0)	< 0.001
	South America	2 (75)	0.7 (0.0–12.1)	2.6 (0.0–18.6)	7.6 (0.0–30.2)	8.4 (0.0–31.7)	63.4 (32.1–93.7)	17.3 (0.0–46.7)	83.5 (31.5–96.0)	0.014
Healthy patients		11 (2776)	0.5 (0.0–3.1)	1.2 (0.0–4.6)	4.1 (0.2–10.9)	9.7 (2.8–18.2)	79.6 (62.7–85.3)	4.9 (0.4–11.9)	97.5 (96.6–98.2)	< 0.001
Hyperparathyroidism patients—overall		10 (1910)	0.4 (0.0–2.3)	0.8 (0.0–3.4)	1.4 (0.0–4.4)	8.4 (3.1–15.4)	82.7 (72.0–89.2)	6.3 91.8–12.7)	94.1 (91.1–96.1)	< 0.001
Secondary hyperparathyroidism patients		9 (1868)	0.3 (0.0–2.4)	0.7 (0.0–3.2)	1.2 (0.0–4.4)	8.8 (3.0–16.5)	82.7 (70.8–89.4)	6.4 (1.5–13.3)	94.7 (91.9–96.6)	< 0.001
MEN 1		2 (129)	0.4 (0.0–5.7)	0.4 (0.0–5.7)	0.4 (0.0–5.7)	0.4 (0.0–5.7)	78.8 (59.2–95.2)	19.6 (4.8–40.8)	83.5 (31.6–96.0)	0.014

*PPE* pooled prevalence estimate, *PTG* parathyroid gland

**Table 3:** Number of parathyroid glands per patient (0–11)

Subgroup	Number of studies (number of patients)	PPE of patients with up to 4 PTG: % (95% CI)	PPE of patients with 5 PTG: % (95% CI)	PPE of patients with 6 PTG: % (95% CI)	PPE of patients with 7 PTG: % (95% CI)	PPE of patients with 8 PTG: % (95% CI)	PPE of patients with 9 PTG: % (95% CI)	PPE of patients with 10 PTG: % (95% CI)	PPE of patients with 11 PTG: % (95% CI)	*I*^2^: % (95% CI)	Cochran’s Q, *p* value
Overall^a^	24 (6910)	93.6 (91.9–97.1)	4.5 (2.4–7.3)	0.7 (0.0–1.8)	0.3 (0.0–1.1)	0.2 (0.0–1.0)	0.2 (0.0–0.9)	0.2 (0.0–0.9)	0.2 (0.0–1.0)	95.2 (93.8–96.2)	< 0.001
Healthy patients	6 (1191)	91.9 (88.9–95.9)	5.2 (2.6–8.7)	1.7 (0.3–3.9)	0.3 (0.0–1.2)	0.3 (0.0–1.2)	0.2 (0.0–1.0)	0.2 (0.0–1.0)	0.3 (0.0–1.2)	72.3 (36.0–88.0)	0.003
Secondary hyperparathyroidism patients	2 (1392)	86.5 (81.5–90.8)	12.1 (8.0–16.8)	1.0 (0.0–2.9)	0.4 (0.0–1.4)	0.0 (0.0–0.5)	0.0 (0.0–0.5)	0.0 (0.0–0.5)	0.0 (0.0–0.5)	84.2 (35.0–96.2)	0.012

*PPE* pooled prevalence estimate, *PTG* parathyroid gland

^a^Overall—includes healthy, MEN1, primary, and secondary hyperparathyroidism patients

### Location of parathyroid glands

Overall analysis of eight studies (*n* = 7529 PTG) showed that 15.9% of PTG are present in ectopic locations, with 11.6% (95% CI 5.1–19.1) in the neck and 4.3% (95% CI 0.7–9.9) in mediastinum (Table 4). Five studies (*n* = 4324 PTG) of healthy patients and three studies (*n* = 1046 PTG) of HPT patients revealed that 94.7% and 82.5% of PTG are localized along the posterior wall of thyroid glands in or within close proximity to their orthotopic locations.

**Table 4:** Location of parathyroid glands

Subgroup	Number of studies (number of PTG)	Orthotopic: % (95% CI)	Neck: % (95% CI)	Mediastinum: % (95% CI)	*I*^2^: % (95% CI)	Cochran’s Q, *p* value
Overall	8 (7529)	84.1 (71.9–89.1)	11.6 (5.1–19.1)	4.3 (0.7–9.9)	98.6 (98.1–99.0)	< 0.001
Healthy	5 (4324)	94.7 (87.4–98.6)	2.7 (0.0–7.9)	2.7 (0.0–7.0)	93.6 (88.0–96.6)	< 0.001
Hyperparathyroidism patients	3 (1046)	82.5 (67.5–92.3)	12.3 (3.4–24.5)	5.2 (0.1–14.8)	95.2 (89.2–97.8)	< 0.001

The subgroup analysis (nine studies, *n* = 435 PTG localized in the neck) showed that 31.4% (95% CI 0.6–63.8) of ectopic PTG in the neck are localized in retroesophageal/paraesophageal space, followed by 20.3% (95% CI 0.0–48.2) in the thyroid gland, 17.7% (95% CI 0.0–44.8) in the carotid sheath, 17.0% (95% CI 0.0–43.9) in the thyrothymic ligament, 5.1% (95% CI 0.0–25.1) in the tracheoesophageal groove, and 8.4% in other locations (thyroid cartilage, retropharyngeal space, adjacent to hyoid bone). The subgroup analysis of mediastinal PTG revealed that majority were located in the thymus.

## Discussion

A careful resection of PTG in patients with HPT and preservation of these glands during thyroidectomies and other surgeries of the neck requires thorough knowledge on the anatomy and possible locations of PTGs. Instead of basing anatomical knowledge on epidemiological studies with small sample sizes, recent years have introduced meta-analysis as a powerful tool for elucidating the complex and variable human anatomy through an evidence-based approach [32]. Therefore, this study aimed to systematically analyze available data on the prevalence and location of parathyroid glands utilizing meta-analysis.

The main findings of this study showed that majority of both healthy and HPT patients have four PTG. However, almost 19% of patients have fewer or more than four PTG. The prevalence of PTG is of particular interest for surgeons treating patients with SHPT. Those with SHPT due to advanced chronic kidney disease who are refractory to initial treatment with vitamin D analogs and calcimimetics are qualified for surgery [1]. A recent meta-analysis has shown the superiority of surgery over pharmacological treatment in terms of all-cause and cardiovascular mortality [1]. Currently, two approaches are advised—subtotal parathyroidectomy, which involves resection of most glands with partial in situ resection of the last one, and total parathyroidectomy with or without heterotopic autotransplantation of a small section of one gland into brachialis muscle. The study by Anderson et al. [33] reported no evident advantage of either methods in terms of complication rates, readmission, and 30-day mortality. However, the success of both types of surgeries depends strongly on identification of all PTG. Our results showed that surgeons should expect that up to 1 in 15 patients will have five or more PTG. Moreover, one in six patients will have ectopically localized glands—with majority located in retroesophageal/paraesophageal space, thyroid, carotid sheath, thyrothymic ligament, and thymus. Subgroup analyses showed that most patients with supernumerary glands have five or six PTG and very rarely seven or more PTG. No differences were observed between healthy and HPT patients and between cadaveric and intraoperative studies.

The clinical picture of PHPT has evolved over time. The classic presentation of PHPT includes decreased bone density with accompanying fractures, renal manifestations such as nephrolithiasis, nephrocalcinosis, renal dysfunction, or neurocognitive symptoms, such as low energy, difficulty with concentration and memory, depression, and anxiety [34]. However, due to widespread laboratory screening, most patients are diagnosed with PHPT in early stage when symptoms are rare or non-evident [34]. While it is commonly agreed that symptomatic patients with PHPT should undergo surgery, the recommendations for operative treatment for asymptomatic patients have shifted. A study of natural history of PHPT by Rubin et al. [35] showed that in more than third of the patients who did not undergo surgery, PHPT progressed over 15 years of follow-up and those patients eventually met criteria for surgical treatment. The current guidelines recommend parathyroidectomy for asymptomatic patients based on age, serum calcium level, bone density, and kidney function [36]. However, it is important to note that parathyroidectomy should be considered for all PHPT patients, as surgery is the only cure for PHPT and is more cost-effective than pharmacological treatment or observation [37].

Preoperative identification of enlarged and hyperfunctioning PTG is of crucial importance for successful minimally invasive procedures. While normal PTG are relatively difficult to visualize in USG, adenomatous, hyperplastic, or carcinomatous glands are more visible. The sensitivity of USG to identify enlarged PTG preoperatively reaches 70–100% [38, 39]. ^99m^Tc-sestamibi imaging of parathyroid glands can be used as an alternative or in conjunction with USG. This technique has a reported sensitivity of 54–100% [40, 41]. When compared, neither of the methods has been shown to be superior in detecting abnormal PTG when carried out by an experienced radiologist [42]. A CT scan can provide additional information on the localization of PTG with a sensitivity ranging from 40 to 86% depending on the size of the gland [43]. It can be useful in detecting missed PTG in sestamibi imaging, thus guiding focused exploration [44]. However, all methods show diminished sensitivity in detecting PTG in rare ectopic locations, such as intrathyroid PTG. Our study showed that 20.3% of ectopic PTG in the neck are located in the thyroid gland. Surgeons should take caution for any intrathyroid PTG during parathyroidectomy, especially in the cases where bilateral neck exploration fails to identify a missing gland or intraoperative PTH assay does not show a decrease of PTH levels of more than 50%.

This meta-analysis was limited by high heterogeneity during the analyses. Despite conducting subgroup analyses to probe the source of heterogeneity, it persisted throughout the study. Additionally, there was a wide discrepancy in the method of reporting the number of glands per patient (especially with supernumerary glands and ectopic glands). The number of studies analyzing morphology of the PTG was also limited.

## Conclusion

Most healthy and hyperparathyroidism patients have four parathyroid glands, but about 20% present with fewer or more glands. The number of ectopic glands reaches 16% and is located in the neck or mediastinum. Surgeons should be aware of the most frequent potential locations of ectopic PTG to assure successful surgery and treatment.

## Electronic supplementary material

Supplement 1 Preferred Reporting Items for Systematic Reviews and Meta-Analyses (*PRISMA*) checklist (DOC 63 kb)

## References

[1] Apetrii M, Goldsmith D, Nistor I, Siriopol D, Voroneanu L, Scripcariu D, Vervloet M, Covic A (2017). Impact of surgical parathyroidectomy on chronic kidney disease-mineral and bone disorder (CKD-MBD)—a systematic review and meta-analysis. PLoS One.

[2] Akerström G, Malmaeus J, Bergström R (1984). Surgical anatomy of human parathyroid glands. Surgery.

[3] Policeni BA, Smoker WRK, Reede DL (2012). Anatomy and embryology of the thyroid and parathyroid glands. Semin Ultrasound CT MR.

[4] Higgins JP, Green S (2008). Cochrane handbook for systematic reviews of interventions.

[5] Henry BM, Tomaszewski KA, Walocha JA (2016). Methods of evidence-based anatomy: a guide to conducting systematic reviews and meta-analysis of anatomical studies. Ann Anat.

[6] Abboud B, Sleilaty G, Braidy C, Ghorra C, Abadjian G, Tohme C, Noun R, Sarkis R (2008). Enlarged parathyroid glands discovered in normocalcemic patients during thyroid surgery. Am J Surg.

[7] Alveryd A (1968). Parathyroid glands in thyroid surgery. I. Anatomy of parathyroid glands. II. Postoperative hypoparathyroidism—identification and autotransplantation of parathyroid glands. Acta Chir Scand.

[8] Arnalsteen L, Proye C (2003). Surgery of hyperparathyroidism and of its potential recurrence in the MEN I setting. Ann Chir.

[9] Botelho JBL, Cançado ARS, de Sousa EA (2004). Importância anatomocirúrgica das características macroscópicas, localização e suprimento vascular das glândulas paratireóides cervicais. Rev Col Bras Cir.

[10] Butterworth PC, Nicholson ML (1998). Surgical anatomy of the parathyroid glands in secondary hyperparathyroidism. J R Coll Surg Edinb.

[11] de Andrade JSC, Mangussi-Gomes JP, da Rocha LA, Ohe MN, Rosano M, das Neves MC, Santos RO (2014). Localization of ectopic and supernumerary parathyroid glands in patients with secondary and tertiary hyperparathyroidism: surgical description and correlation with preoperative ultrasonography and Tc99m-Sestamibi scintigraphy. Braz J Otorhinolaryngol.

[12] Edis AJ, Levitt MD (1987). Supernumerary parathyroid glands: implications for the surgical treatment of secondary hyperparathyroidism. World J Surg.

[13] Ghandur-Mnaymneh L, Cassady J, Hajianpour MA (1986). The parathyroid gland in health and disease. Am J Pathol.

[14] Gilmour JR (1938). The gross anatomy of the parathyroid glands. J Pathol Bacteriol.

[15] Gomes EMS, Nunes RC, Lacativa PGS, Almeida MH, Franco FM, Leal CTS, Patrício Filho PJM, Farias MLF, Gonçalves MDC (2007). Ectopic and extranumerary parathyroid glands location in patients with hyperparathyroidism secondary to end stage renal disease. Acta Cir Bras.

[16] Heinbach WF (1933). A study of the number and location of the parathyroid glands in man. Anat Rec.

[17] Hellman P, Skogseid B, Öberg K (1998). Primary and reoperative parathyroid operations in hyperparathyroidism of multiple endocrine neoplasia type 1. Surgery.

[18] Hibi Y, Tominaga K, Uchida K (2002). Cases with fewer than four parathyroid glands in patients with renal hyperparathyroidism at initial parathyroidectomy. World J Surg.

[19] Hojaij F, Vanderlei F, Plopper C, Rodrigues CJ, Jácomo A, Cernea C, Oliveira L, Marchi L, Brandão L (2011). Parathyroid gland anatomical distribution and relation to anthropometric and demographic parameters: a cadaveric study. Anat Sci Int.

[20] Kawata R, Kotetsu L, Takamaki A, Yoshimura K, Takenaka H (2009). Ultrasonography for preoperative localization of enlarged parathyroid glands in secondary hyperparathyroidism. Auris Nasus Larynx.

[21] Lappas D, Noussios G, Anagnostis P, Adamidou F, Chatzigeorgiou A, Skandalakis P (2012). Location, number and morphology of parathyroid glands: results from a large anatomical series. Anat Sci Int.

[22] Milas M, Wagner K, Easley KA, Siperstein A, Weber CJ (2003). Double adenomas revisited: nonuniform distribution favors enlarged superior parathyroids (fourth pouch disease). Surgery.

[23] Nanka O, Sedý J, Vítková I, Libánský P, Adámek S (2006). Surgical anatomy of parathyroid glands with emphasis on parathyroidectomy. Prague Med Rep.

[24] Numano M, Tominaga Y, Uchida K, Orihara A, Tanaka Y, Takagi H (1998). Surgical significance of supernumerary parathyroid glands in renal hyperparathyroidism. World J Surg.

[25] Okada M, Tominaga Y, Yamamoto T, Hiramitsu T, Narumi S, Watarai Y (2016). Location frequency of missed parathyroid glands after parathyroidectomy in patients with persistent or recurrent secondary hyperparathyroidism. World J Surg.

[26] Pacini P, Biondi G, Cicchi P (1983). The number and topographical location of parathyroid glands in man. Arch Ital Anat Embriol.

[27] Périé S, Fessi H, Tassart M, Younsi N, Poli I, St Guily JL, Talbot JN (2005). Usefulness of combination of high-resolution ultrasonography and dual-phase dual-isotope iodine 123/technetium Tc 99m sestamibi scintigraphy for the preoperative localization of hyperplastic parathyroid glands in renal hyperparathyroidism. Am J Kidney Dis.

[28] Pool EH, Falk HC (1916). Concerning the surgrical anatomy of the thyroid with special reference to the parathyroid glands. Ann Surg.

[29] Praženica P, O’Keeffe L, Holý R (2015). Dissection and identification of parathyroid glands during thyroidectomy: association with hypocalcemia. Head Neck.

[30] Pyrtek L, Painter RL (1964). An anatomic study of the relationship of the parathyroid glands to the recurrent laryngeal nerve. Surg Gynecol Obstet.

[31] Wang C (1976). The anatomic basis of parathyroid surgery. Ann Surg.

[32] Tomaszewski KA, Henry BM, Pękala PA, Standring S, Tubbs RS (2018). The new frontier of studying human anatomy: introducing evidence-based anatomy. Clin Anat.

[33] Anderson K, Ruel E, Adam MA (2017). Subtotal vs. total parathyroidectomy with autotransplantation for patients with renal hyperparathyroidism have similar outcomes. Am J Surg.

[34] Stephen AE, Mannstadt M, Hodin RA (2017). Indications for surgical management of hyperparathyroidism. JAMA Surg.

[35] Rubin MR, Bilezikian JP, McMahon DJ (2008). The natural history of primary hyperparathyroidism with or without parathyroid surgery after 15 years. J Clin Endocrinol Metab.

[36] Bilezikian JP, Brandi ML, Eastell R, Silverberg SJ, Udelsman R, Marcocci C, Potts JT (2014). Guidelines for the management of asymptomatic primary hyperparathyroidism: summary statement from the fourth international workshop. J Clin Endocrinol Metab.

[37] Wilhelm SM, Wang TS, Ruan DT, Lee JA, Asa SL, Duh QY, Doherty GM, Herrera MF, Pasieka JL, Perrier ND, Silverberg SJ, Solórzano CC, Sturgeon C, Tublin ME, Udelsman R, Carty SE (2016). The American Association of Endocrine Surgeons Guidelines for definitive management of primary hyperparathyroidism. JAMA Surg.

[38] Carneiro-Pla D (2009). Recent findings in the use of intraoperative parathyroid hormone monitoring in parathyroid disease. Curr Opin Oncol.

[39] Gilat H, Cohen M, Feinmesser R, Benzion J, Shvero J, Segal K, Ulanovsky D, Shpitzer T (2005). Minimally invasive procedure for resection of a parathyroid adenoma: the role of preoperative high-resolution ultrasonography. J Clin Ultrasound.

[40] Borley NR, Collins REC, O’Doherty M, Coakley A (1996). Technetium-99m sestamibi parathyroid localization is accurate enough for scan-directed unilateral neck exploration. Br J Surg.

[41] McHenry CR, Lee K, Saadey J, Neumann DR, Esselstyn CB Jr (1996). Parathyroid localization with technetium-99m-sestamibi: a prospective evaluation. J Am Coll Surg.

[42] Haber RS, Kim CK, Inabnet WB (2002). Ultrasonography for preoperative localization of enlarged parathyroid glands in primary hyperparathyroidism: comparison with (99m) technetium sestamibi scintigraphy. Clin Endocrinol.

[43] Mohebati A, Shaha AR (2012). Imaging techniques in parathyroid surgery for primary hyperparathyroidism. Am J Otolaryngol.

[44] Harari A, Zarnegar R, Lee J, Kazam E, Inabnet WB, Fahey TJ (2008). Computed tomography can guide focused exploration in select patients with primary hyperparathyroidism and negative sestamibi scanning. Surgery.

